# Si doped T6 carbon structure as an anode material for Li-ion batteries: An *ab initio* study

**DOI:** 10.1038/srep37822

**Published:** 2016-11-28

**Authors:** A. Rajkamal, E. Mathan Kumar, V. Kathirvel, Noejung Park, Ranjit Thapa

**Affiliations:** 1 Department of Physics and Nanotechnology, SRM University, Kattankulathur 603203, Tamil Nadu, India; 2 SRM Research Institute, SRM University, Kattankulathur 603203, Tamil Nadu, India; 3 Department of Physics, Center for Multidimensional Carbon Materials, Institute for Basic Science (IBS), Ulsan 689-798, Republic of Korea

## Abstract

First-principles calculations are performed to identify the pristine and Si doped 3D metallic T6 carbon structure (having both sp^2^ and sp^3^ type hybridization) as a new carbon based anode material. The π electron of C_2_ atoms (sp^2^ bonded) forms an out of plane network that helps to capture the Li atom. The highest Li storage capacity of Si doped T6 structure with conformation Li_1.7_Si_1_C_5_ produces theoretical specific capacity of 632 mAh/g which substantially exceeding than graphite. Also, open-circuit voltage (OCV) with respect to Li metal shows large negative when compared to the pristine T6 structure. This indicates modifications in terms of chemical properties are required in anode materials for practical application. Among various doped (Si, Ge, Sn, B, N) configuration, Si doped T6 structure provides a stable positive OCV for high Li concentrations. Likewise, volume expansion study also shows Si doped T6 structure is more stable with less pulverization and substantial capacity losses in comparison with graphite and silicon as an anode materials. Overall, mixed hybridized (sp^2^ + sp^3^) Si doped T6 structure can become a superior anode material than present sp^2^ hybridized graphite and sp^3^ hybridized Si structure for modern Lithium ion batteries.

The rechargeable types of Lithium-ion batteries (LIB’s) are becoming incredibly popular due to light weight mobile power source, self-discharge, mainly owing to their high energy density and fast charge/discharge rate. To bring this application for heavier systems, such as electric vehicles (EVs), plug-in hybrid electric vehicles (PHEV) and hybrid electric vehicles (HEV), a significant improvements in the specific energy capacity is required^1^,^2^,^3^,^4^,^5^. The energy capacity is adjusted mainly through the engineering of anode materials which is the most essential key component for electrochemical performances of the LIB’s^6^. Ever since industrial production of LIB’s, graphitic carbon anode materials are the most actively studied and it can reversibly accommodate Lithium (Li) ions between sp^2^-bonded layers. In the perspective of an ideal anode, not only the capacity, but also the structural robustness over the charge cycle i.e, charging and discharging processes are also of great significance^7^. Furthermore, the kinetics related to Li diffusion time determines the quality of the electrode, and adjustment of microscopic particle sizes of graphitic materials are considered in this regard.

Compared with anodic capacity 3860 mAh/g^8^ of metal Li, the graphite (a sp^2^ hybridized material) as an anode is quite low: a maximal LiC_6_ conformation can produce 372 mAh/g^9^. Nevertheless, Li intercalated graphitic anodes is proved that has low operating voltage, remarkable interfacial stability, and resistivity against the dendrite growth and successful fabirication^10^. To achieve a higher capacity anode, silicon (Si) nanostructures (a sp^3^ hybridized material) are considered the most in which the theoretical capacity could reach upto 4200 mAh/g^11^,^12^,^13^. This higher capacity of Si nanostructure is because of non-layered crystal structural arrangement^13^. The most abundant, inexpensive and eco-friendly Si anode material has discharge (lithiation) potential of 0.4 V vs. Li/Li^+^. However, to date, pristine Si anode materials are not so practical: few cycle of charging/discharging process led to the volume expansion by 400%, results in pulverization and substantial capacity losses^12^. These are the reason why researchers still in search for a derivatives of carbon (C) materials in the pursuit of a better anodic material.

Among carbon derivatives, graphene also proves that has good safety, conductivity^14^ and cycle stability. But the specific capacity is limited due to presence of ordered stacking of graphene layers which gives one intercalated layer of Li per graphene sheets. Raman spectra studies reveal that the capacity of Li adsorbed on a single layer of graphene is lower than that of few layers of graphenes^15^. The Li atom has low affinity towards pristine graphene, that leads to agglomeration caused by strong Li-Li interaction^16^,^17^. Still, Theoreticians are searching for other carbon allotrope based on sp^2^-bonded carbon networks. Zang *et al*. reported that graphyne (one-atom-thick planar sheets of sp and sp^2^ bonded C atoms) material which is 2D carbon allotropes with larger pores than hexagon graphene can leads to Li_1.5_C_6_ conformation^18^. Another derivatives, namely graphenylene (2D carbon allotrope consisting of four and six rings of non-delocalized sp^2^-carbon structure with larger pores than graphene) which has nonzero bandgap and well-delocalized frontier orbitals, is suggested to achieve a higher theoretical specific capacities of about 1116 mAh/g, corresponding to Li_3_C_6_ conformation^19^. This obviously indicates that carbon materials can provide more rooms in the pursuit of future anodic materials for LIBs. In this regard, recently report by Zhang *et al*. is greatly noteworthy: a unique phases of three dimensional (3D) metallic with a high symmetric space group (P4_2_/mmc) structure that has a simple tetragonal primitive cell containing six C atoms (called T6 carbon) which are interlocked through hexagonal rings. The structure is coined as T6 carbon (a mixed sp^2^ and sp^3^ hybridized material) and its thermodynamic and mechanical stability are well assessed^20^. Since, metallic conductivity of T6 carbon are well known, here we mostly focused on the Li adsorption and intercalation mechanism in the view of novel carbon-based anode material.

In this work, we carry out calculations using density functional theory (DFT) to identify Si doped T6 structure (a mixed sp^2^ and sp^3^ hybridized material) as a new type of anode material for LIB’s. The Li adsorption on pristine T6 (100) surface studies are in detail. We explore the electronic structures to rationalize the nature of surface interaction between Li atom and nearest neighbour C atoms of T6 (100) surfaces. A comprehensive study of density of states (DOS) and electron localization function (ELF) are studied for Si doped T6 to analyse bonding nature of C-Si, Li-C and Li-Si. Eventually, our calculated results show that the open-circuit voltages (OCV) and formation energies (ΔE_f_) are in the desirable range (as required for anode materials) for the case of Si doped T6 structure. Here, we discuss overall prospect of T6 carbon as anode material in terms of the theoretical specific capacity and resistivity against Li clustering.

## Results and Discussions

### Electronic structure of T6 carbon

**Figure 1:**
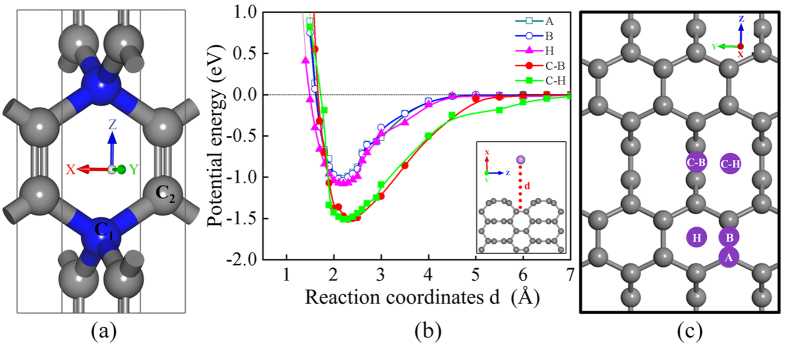
(**a**) Simple tetragonal primitive cell of T6 carbon. The blue coloured carbon atoms are sp^3^ hybridized and denotes as C_1_. Grey coloured C_2_ carbon atoms are sp^2^ hybridized. (**b**) Potential energy curve (PEC) for Li adsorbed on five different sites of T6 (100) surface. The green, red, pink, blue and dark green curves represent PEC considering Centre-Hollow (C-H), Centre-Bridge (C-B), Hex-Hollow (H), CC-Bridge (B) and Atop (A) sites. The reaction coordinate ‘d’ is chosen to be a vertical distance of Li atom from each site of the adsorbed T6 (100) surface, (**c**) the different sites are shown using the top view of T6 (100) surface. The grey and violet balls denote the C and Li atom respectively.

The optimized crystal structure of the unique carbon allotrope of T6 carbon is shown in Fig. 1(a). The unit cell of T6 carbon consists of two chemically non-equivalent atomic Wyckoff positions, 2f (1/2, 1/2, 1/4) and 4i (1/2, 0, 0.1118) sites occupied by the sp^3^ (C_1_, coloured blue) and sp^2^ (C_2_, coloured grey) hybridized C atoms, respectively. The unit cell consists of six C atoms: two C_1_ atoms and four C_2_ carbon atoms. The C_1_-C_2_ bond distance is found to be longer than that of the C_2_-C_2_ by about 0.2 Å, in agreement with difference between sp^3^ and sp^2^ bond lengths. Zhang *et al*., reported that electronic band structure shows the metallic behaviour of T6 carbon. It is found from PDOS plot that π electron of the C_2_ atoms (sp^2^ hybridized) is mainly contribute to occupied state at the Fermi level, whereas the electrons of C_1_ (sp^3^ hybridized) atoms have no contribution near the Fermi level and no role in the metallicity of T6 carbon. In addition, the charge density comes from C_2_ atoms and electrons in their π orbitals form a delocalized network along the direction of (001) and (100) surface of T6.

### Lithium adsorption on T6 (100)

In addition to the metallicity, Li ionic adsorption and migration properties need to consider in detail for an anode material. We treat T6 (100) surface and created (3 × 2 × 1) supercell with three layers slab which contains 108 carbon atoms. We also consider the T6 (001) slab which contains 81 carbon atoms of (3 × 3 × 1) supercell (see Fig. S1 of supplementary information) to check the prospect of other facet. But we find that the energy per atom of T6 (001) and T6 (100) slabs are −153.85 eV and −154.21 eV respectively which shows that T6 (100) surface is more stable compared to T6 (001) surface by energy of 360 meV per atom. A vacuum slab of 20 Å is placed in the supercell to exclude spurious interaction between replicas present in the periodic boundary conditions. For simplicity we consider the vacuum space above T6 (100) plane for this work, actually more complex solid electrolyte interface is present (which is not focus of this work). The adsorption energy (ΔE_ad_) of Li on T6 (100) is calculated and it is defined as follows,


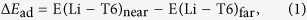


where E(Li − T6)_near_ is the total energy of Li adsorbed on T6 (100) and E(Li − T6)_far_ is the total energy of the system in which Li placed about 6 Å distance from the surface of T6 (100). We consider five different sites (see Fig. S2 of supplementary information): Atop (A) site (directly above the carbon), C-C Bridge (B) site (above midpoint of C-C bond), Hex-Hollow (H) site (above the centre of hexagonal ring), Centre-Bridge (C-B) site (above centre of bridge of two hexagonal rings), Centre-Hollow (C-H) site (between bridges of two hexagonal rings) in T6 (100). The calculated ΔE_ad_ are −1.02, 04, −1.08, −1.51 and −1.54 eV at A, B, H, C-B and C-H sites respectively, listed in Table 1. These results reveal that among all sites C-B & C-H have higher ΔE_ad_ compared to other sites. It indicates, these two sites are more active. Because, the surface of T6 (100) in which C_2_ atoms are bonded via sp^2^ hybridization (no dangling bond) by network of out of plane π electron and C_1_ atoms are now have three bonded network with forth one as dangling bond. So, in this surface the Li atoms mostly make bond with the surface C_2_ atom via s- π interaction or bond with C_1_ atom via s-σ interaction. Obviously, the Li adsorption onto C-B & C-H sites consists of the Li binding with C_1_ atoms that makes s-σ interaction. In the other sites A, B and H are consist C_2_ atoms that makes s-π weaker interaction than s-σ interaction. Further, these results are verified by calculating the potential energy curves for five sites [see Fig. 1(c)] of T6 (100) are shown in Fig. 1(b). Here, we consider Li atom is displaced perpendicular to the surfaces of T6 (100) as denoted by the dashed line [see inset image of Fig. 1(b)]. From this potential energy plot, it is found that the interaction between C-Li atom is negligible when Li atoms are far apart from the surface by about 6 Å. As Li atoms get closer to the surface, the interaction increases between C-Li showing the strong Coulombic attraction. The depth of the minimum in the potential energy curves represents the values of ΔE_ad_ of Li atoms on T6 (100). When the Li atoms get very much close to the surface, the steep in curves rises in potential energy which represents a repulsive force between C-Li atoms.

### Charge density and partial density of states: Li to Li^+^

**Figure 2:**
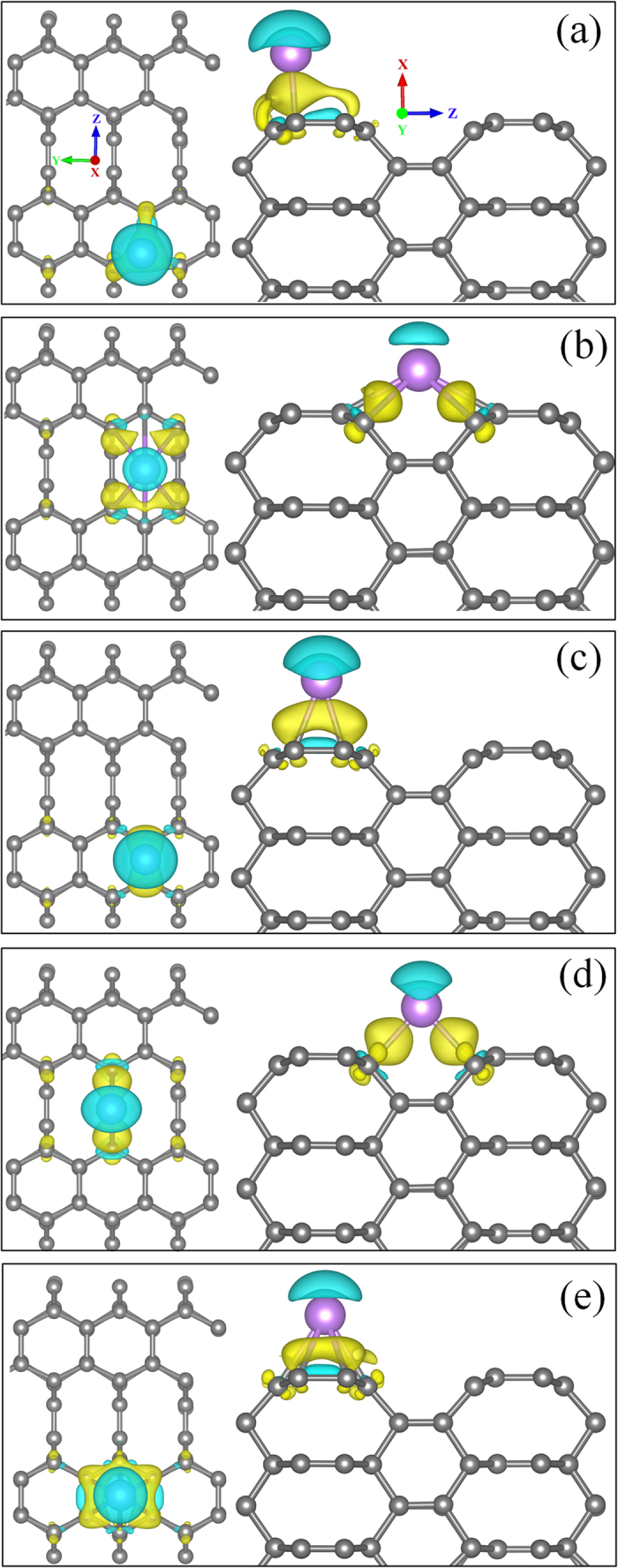
Charge density difference for Li adsorption on (**a**) Atop, (**b**) Centre-Hollow, (**c**) C-C Bridge, (**d**) Centre-Bridge and (**e**) Hex-Hollow sites of T6 (100) surface. Blue and Yellow lobes correspond to depletion and gain of electronic charge respectively. The isosurface value of 3 × 10^−3^ e Bohr^−3^ is considered for all cases. The grey and violet spheres represent C and Li atom respectively.

To have a better understanding of the nature of binding between Li and C, the difference in charge density is calculated. It is defined as


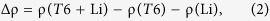


where ρ(T6 + Li), ρ(T6) and ρ(Li), are total charge densities of Li adsorbed T6 (100), pristine T6 (100) and isolated Li atom respectively, with T6 (100) and Li atoms in the exact same positions as they adsorbed in T6 (100). Figure 2(a–e) shows charge density difference for five sites on T6 (100). In all the cases net gain of electronic charges are occupied in the central region between Li and T6 (100) surface and also there is a net loss of electronic charges occupied above the Li atom. The depicted charge is transferred from Li atom to the surface C atoms of T6 (100). Bader charge analysis shows that Li atom completely transfers its one electron to the surface C atoms in all cases and it becomes ionized to Li^+^. This ionic interaction between Li and C atoms can be attributed to charge transfer from Li-2s state to carbon 2p state.

**Figure 3:**
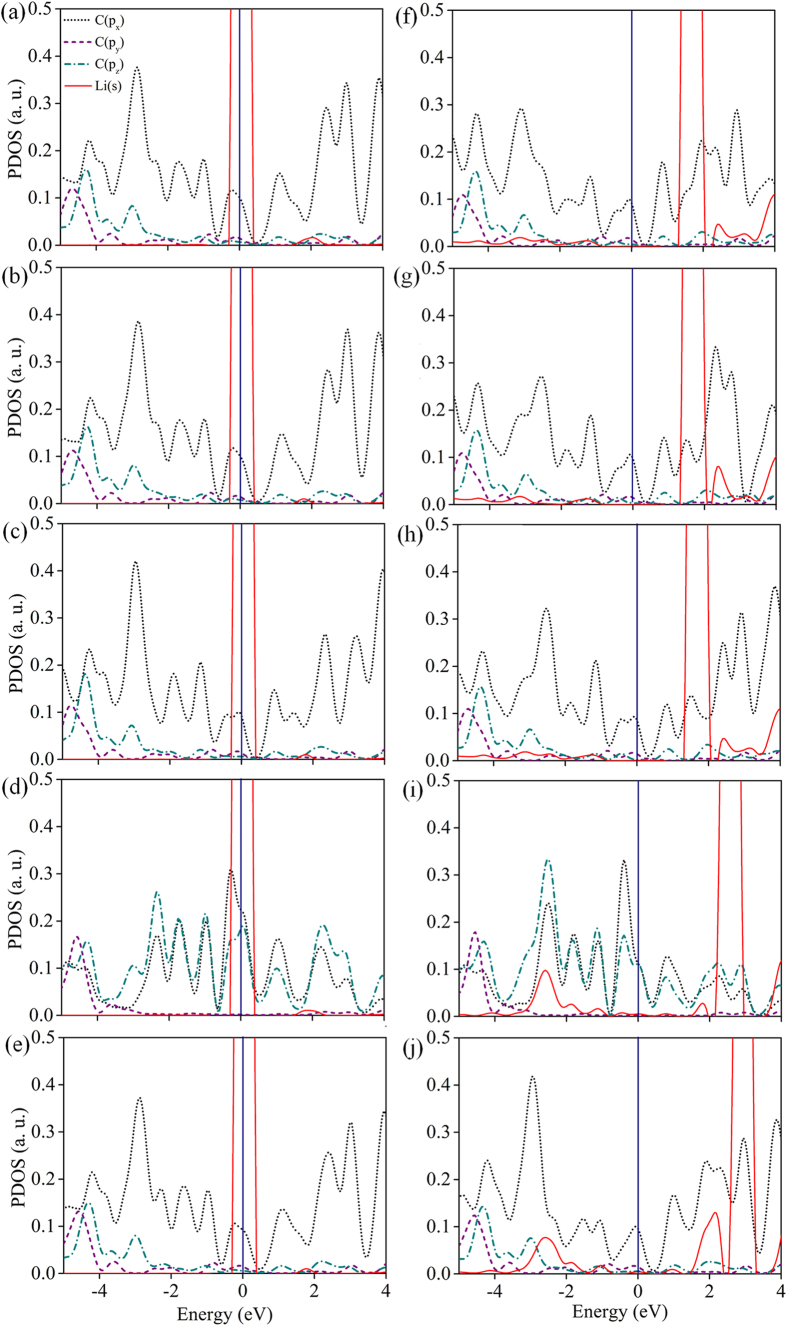
Partial density of states (PDOS) for Li and the surface C atom on which Li sits on. (**a**) The Atop C atom (A), (**b**) C-C Bridge (B), (**c**) Hex-Hollow (H), (**d**) Centre-Bridge (C-B) and (**e**) Centre-Hollow (C-H) sites when the Li atom is placed 6 Å above from the adsorption site. (**f**)–(**j**) The same PDOS for Li and the surface C when Li atom adsorbed on the surface of T6 (100). The red (solid line), black (short dot), violet (short dash), green (short dash dot) indicate PDOS of ‘s’ states of Li and ‘px’, ‘py’, ‘pz’ of C atom respectively and the Fermi level is denoted with blue line.

We further calculated partial density of states (PDOS) to understand Li binding properties from the electronic structure. For all sites of both cases, Li adsorbed and Li placed 6 Å distance from the surface of T6 (100) are shown in Fig. 3. Our computational results show that Li-2s peak lies at the Fermi level in the case of Li placed far from the surface [see Fig. 3(a–e)]. But in the case of Li adsorbed system [see Fig. 3(f–j)], Li-2s peak obviously lies above the Fermi level. These sequences of argument tells that Li atom becomes ionized due to complete charge transfer^21^.

### Diffusion of Li atom across the plane of T6 (100)

The rate of Li^+^ intercalation and de-intercalation largely depends on diffusivity of Li^+^ 22. So, diffusion of Li^+^ across the plane is investigated by considering the energy barriers for the transitions from the most stable adsorption sites (C-B and C-H) to the adjacent sites. ΔE_b_’s are estimated (see Fig. S3 of supplementary information) to about 0.52, 0.06, 0.04 and 0.47 eV for Li^+^ diffusion path from C-H to A, H to A site, H to B site and C-B to B site respectively. The diffusion path from C-H to A and C-B to B site of ΔE_b_ are higher than path from H to A and H to B site due to the higher E_ad_ of Li^+^ with C-H and C-B. For the path from H to A and H to B site, ΔE_b_ are much lower. But, in the case of Si doped T6 surface, ΔE_b_’s are 0.29, 0.1, 0.07 and 0.19 eV for corresponding diffusion path, C-H to A site, H to A site, H to B site and C-B to B site (shown in Fig. S3). Energy barrier from C-H to A and C-B to B sites are remarkably reduced and it makes Li to diffuse much easier.

### Formation energy, Volume expansion and Open circuit voltage

Until now, we concentrate only on the adsorption and kinetics of Li considering the layer structure of T6 (100) surface. These results reveals that T6 (100) surface has reasonable binding energy with low energy barriers against the transport of Li^+^ across the plane, which are thought to be advantageous merit as a candidate for anode material. In practice, during the charging process, Li^+^ diffuse through the electrolyte and intercalate into the anode material. Thus, it is not only the surface properties but the intercalation of Li deep into the anode material is more importance. In order to address these issues, we evaluate the lithium storage capacity in relaxed structure of bulk T6 carbon. In our calculation we used (2 × 2 × 2) supercell of T6, consisting of 48 carbon atoms.

**Figure 4:**
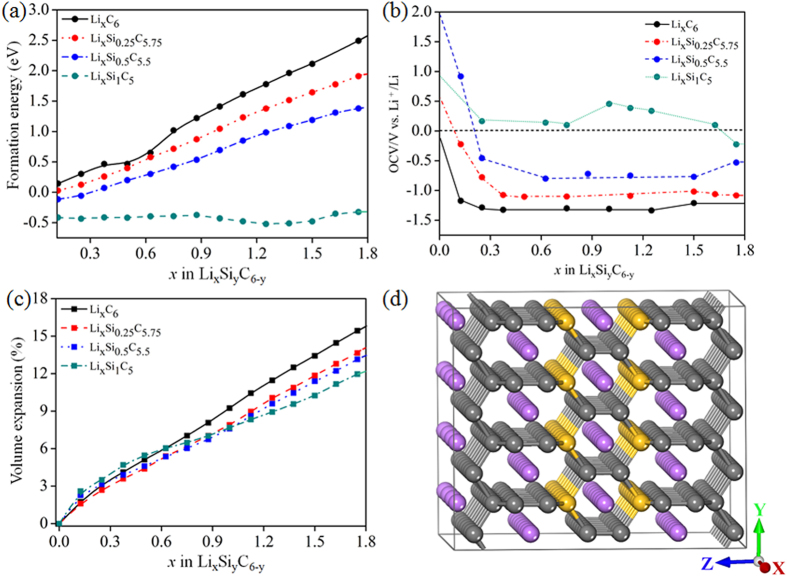
Estimated value of (**a**) Formation Energy ΔE_f_, (**b**) OCV, and (**c**) volume expansion for pristine and Si doped T6 (Li_x_Si_y_C_6-y_) as a function of the Li (x) and Si (y) concentration. (**d**) The optimized structure of Li intercalated Si doped T6 for the conformation Li_1.75_Si_1_C_5_. The grey, yellow and violet spheres are interposing C, Si and Li atoms respectively. The lines serve as guide to the eye. In (**b**), the horizontal dashed line is minimum frontier line for OCV. In (**d**), all Li atoms depict as violet balls positioned in the most stable C-H site.

We studied the stability and prospect of Li intercalation in T6 structure considering the conformation of Li_x_C_6_, we evaluated the formation energy (ΔE_f_) by,


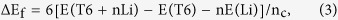


where E(T6 + nLi) is the total energy of n number Li atom/s intercalated T6 structure, E(T6) is the total energy of T6, E(Li) is the total energy of a Li atom in the form of bulk with body-centred cubic lattice, n is the number of intercalated Li atom and n_c_ is the number of carbon atoms in T6 structure. Therefore, x = 6n/n_c_ is the concentration of Li^+^. The variation of ΔE_f_ for Li intercalated material Li_x_C_6_ as a function of x is shown in Fig. 4(a). We found that ΔE_f_ value increases monotonically with increase of Li concentration. For Li_1.75_C_6_ conformation, we observe ΔE_f_ = 2.6 eV, indicates that the process is completely endothermic. In order to get desirable value of ΔE_f_ (negative value, indicates exothermic process for Li intercalation), we screen with various types of dopants such as boron and nitrogen. Due to doping, the structures didn’t change substantially and the systems are quite stable. The nitrogen doped T6 structure (LiN_0.25_C_5.75_) shows higher ΔE_f_ (see Fig. S7(a) of supplementary information) during Li insertion compared to the pristine T6 system (structure of the model is shown in Fig. S6), mainly because of higher electron cloud around the N atom. Whereas, Li intercalation for the concentration of 0.125 in B doped T6 system (Li_0.125_B_0.25_C_5.75_), ΔE_f_ is found to be small positive value [see Fig. S7(a)], but during intercalation of two Li atoms (concentration of 0.25) large structural deformation is taking place. Also in N doped T6 system, after intercalation of few Li atoms, structure became very much unstable. This indicates that, B and N dopants are inappropriate to tune the T6 carbon as an anode material. So, we choose neutral dopants such as silicon (Si), germanium (Ge) and Tin (Sn). Among these systems, Si doped T6 structure with different compositions such as Si_0.25_C_5.75_, Si_0.5_C_5.5_ and Si_1_C_5_ show good stability even after high concentration of Li intercalation [see Fig. 4]. Whereas, in the Ge and Sn doped T6 structures the structural deformation is obtained for Li concentration of 0.875 (Li_0.875_Ge_0.25_C_5.75_) and 0.375 (Li_0.375_Sn_0.25_C_5.75_) respectively. The formation energy is found to be positive (endothermic). The Si doping into T6 at C1 and C2 sites have formation energies 2.04 eV and 2.64 eV respectively. Therefore, doping Si at C1 site is favorable. Li intercalated Si doped T6 atomic structure is shown in Fig. 4(d). Figure 4(a) indicates in midst of all compositions, negative formation energies (i.e exothermic process) for Li intercalation is observed for Si_1_C_5_ structure (mixed hybridized material). It can be seen that for every Li concentration processes are exothermic and value is lower compared to reported LiC_6_ conformation of graphite (sp^2^ hybridized based material) as a host material^23^.

We also plot volume expansion (ΔV) in percentage by comparing the computed equilibrium volumes of the two limiting structures as follow:


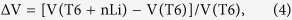


where V(T6) is the volume of the pure and doped T6 structure considered as a reference volume i,e before intercalation of any Li ion and V(T6 + nLi) is the volume of the Li intercalated T6 based structure and n is the increasing concentrations of Li during intercalation. The ΔV is found to be directly proportional to the Li concentrations as shown in Fig. 4(c). The linearity of curve is due to nature of T6 structure (interlocking hexagonal), the volume expansion reaches up to 11.8% at the maximum lithiation (Li_1.75_Si_0.25_C_5_). To be note, for Si doped T6 structures the volume expansion (%) is little lower than the pristine one, indicating Si doped structures are more stable during lithiation. For graphite (layered nature with sp^2^ hybridization) the volume expansion reaches up to 12% for LiC_6_ conformation and for Si (sp^3^ hybridization) as anode material more than 100% of expansion is observed for Li_15_Si_4_ conformation^24^,^25^. Fortunately, our results display that Si doped T6 structure has only 7% in volume expansion at conformation of Li_1_Si_1_C_5_ and gives assurance of safety issues to the standard LIB’s. In the case of B, N, Sn, and Ge doped T6 show high volume expansion (see Fig. S7(c)) even for low Li concentrations (Li_0.125_B_0.25_C_5.75_, Li_0.375_N_0.25_C_5.75_, Li_0.375_Sn_0.25_C_5.75_ and Li_0.875_Ge_0.25_C_5.75_,) compared to the Si doped T6 (Li_0.875_Si_1_C_5.75_). To investigate the usability of Li-dispersed for an anode material, the open-circuit voltage (OCV) is calculated according to


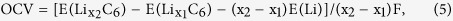


where 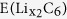 and 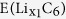 are the total energy of the anode material with the Li concentrations of x_2_ and x_1_, respectively and F is the Faraday’s constant. We consider OCV at the midpoint x i.e. (x_1_ + x_2_)/2 in which OCV is calculated at interval between x_1_ and x_2_. It is clearly seen in Fig. 4(b), the calculated OCV for Li intercalated pristine and different compositions of Si doped T6 structures. For all configurations, minimal energy differences are considered for OCV calculations with chosen concentrations of Li. The OCV value by considering bulk material (without any Li intercalation) as reference is shown in Fig. S5. The OCV values for graphite (reported by Theoretical^25^ and Experimental^26^ group) is decreasing to 0 V vs. Li^+^/Li at LiC_6_ for increasing x values. Among the system considered, for pristine and Si doped T6 structures (Si_0.25_C_5.75_, Si_0.5_C_5.75_), negative value of OCV is obtained. However, Si_1_C_5_ as a host material positive OCV value is observed for maximum range of Li concentration and zero OCV value is noted when the Li concentration reaches around x = 1.7. Other dopants such as B, N, Sn and Ge systems show negative OCV values mostly for all Li concentration [see Fig. S7(b)]. The Si doped T6 (Si_1_C_5_) shows long range positive OCV value which is a good measure for a longer life time of anode materials. But the Li-Li interaction increases for higher Li concentrations. In both pristine and Si doped T6 structure, Li ions prefer to store in the C-H site which has higher binding energy and slightly larger pore than graphite’s hexagonal pore during intercalation (see Fig. S4 of supplementary information). Eventually, we calculate theoretical specific capacity of Li intercalated graphite (LiC_6_) and single wall carbon nanotube (SWCNT) (LiC_2_) structures and are given in Table 2. Theoretical specific capacity of Li_1.7_ Si_1_C_5_ conformation is 632 mAh/g. So the specific capacity of T6 based structure is significantly larger than corresponding values of graphite and graphyne (see Table 2)^24^,^27^,^28^. These affirmative results of adsorption, diffusion, formation energy, volume expansion, OCV and specific capacity of Si doped T6 structure suggesting, it can be good carbon based anode material for LIB’s with long life, safety, high, stability and high specific capacity.

### Density of states and Electron localization function

**Figure 5:**
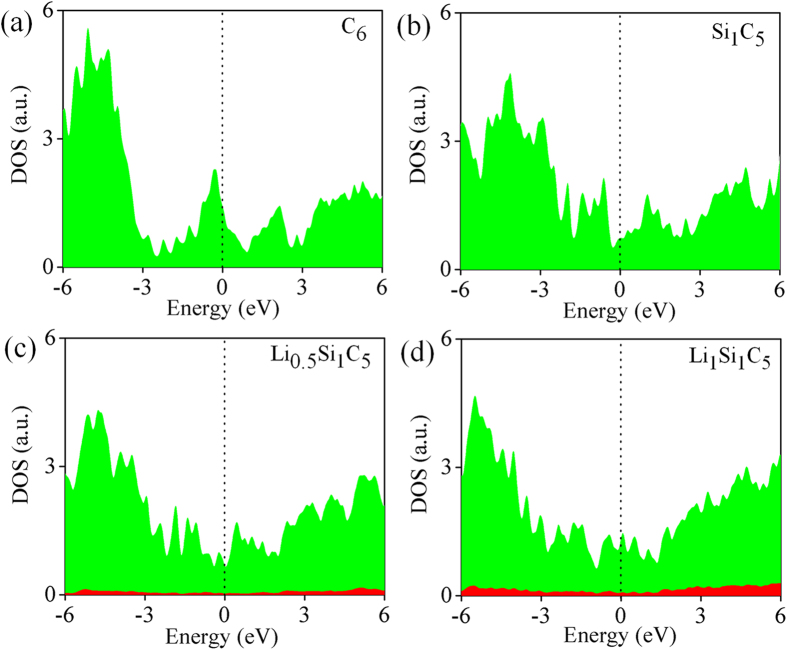
Density of states (DOS) for (**a**) pristine T6 (C_6_), (**b**) Si doped T6 (Si_1_C_5_), Li intercalated Si doped T6 considering (**c**) (Li_0.5_Si_1_C_5_) and (**d**) (Li_1_Si_1_C_5_). (**a**–**d**) Filled green area indicates DOS for ‘p’ states of C atoms, and (**c**,**d**) filled red area represents the DOS for ‘s’ states of Li atoms. The dashed lines indicate the position of Fermi level.

**Figure 6:**
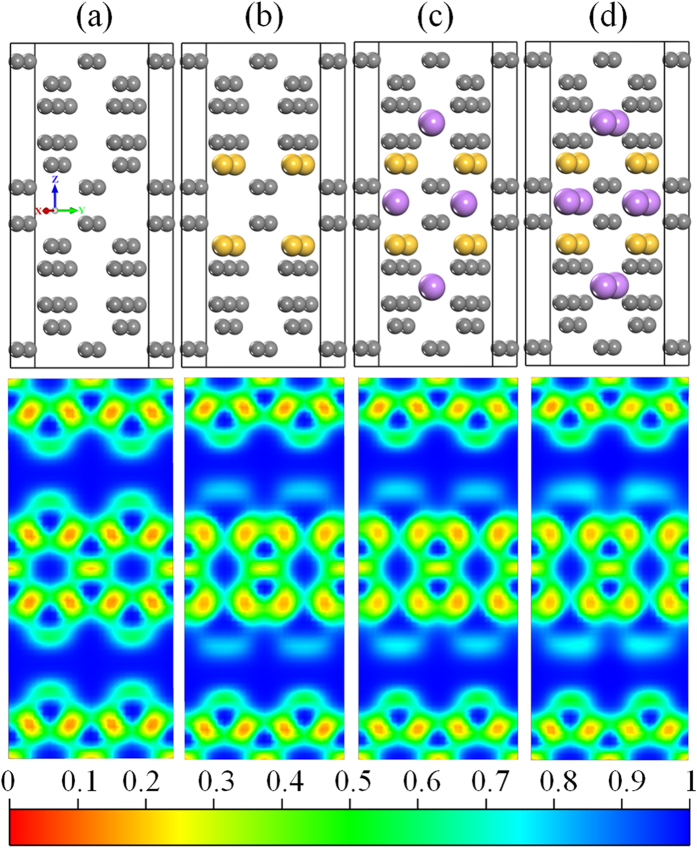
Atomic structures and 2D plot of electron localization function for (**a**) pristine and (**b**) Si doped T6 structure (Si_1_C_5_) (Si doped at C_1_ sites) (**c**) and (**d**) demonstrating the Li intercalated Si doped T6 structure with conformation of (Li_0.5_Si_1_C_5_) and (Li_1_Si_1_C_5_). The grey, yellow and violet spheres are interposing C, Si and Li atoms respectively.

To understand bonding nature of Li-Si and Li-C after Li intercalation, we calculate density of states for pristine, Si doped T6 and Li intercalated Si doped T6 structure. We consider two conformations, Li_0.5_Si_1_C_5_, Li_1_Si_1_C_5_ and are shown in Fig. 5. The metallic behaviour of T6 structure [see Fig. 5(a)] doesn’t change after doping with Si atoms. It is clearly seen from the DOS of Si doped T6 structure [see Fig. 5(b)]. The Fermi level is shifted towards higher energy value^26^, to accommodate the electron transfer from the Li atoms to host structures after intercalation of Li [See Fig. 5(c,d)]. This implies that Li-Si and Li-C bonds are ionic in character. We observe similar results in case of other dopants (B, N, Sn and Ge) can be understood from the DOS plot [see Fig. S8 of supplementary information]. To strengthen above argument, we perform the electron localization function (ELF) study for pristine, Si doped T6 and Li intercalated Si doped T6 i.e. Li_0.5_Si_1_C_5_ and Li_1_Si_1_C_5_ conformations. The covalent interaction between C-C and Si-C are more clearly illustrated from the ELF plot shown in the Fig. 6(a–d). In pristine T6 structure, sp^2^ and sp^3^ bonded C atoms are surrounded by high localization with the value of around 0.8. This clearly indicates that C atoms are bonded covalently^29^ (denoted by red-yellow contours) and the pore regions have less localization (indicated by blue contours). But Si doped T6 case, small reduction in the localization of charge between the Si and C atoms is observed, indicating less covalent character of Si-C bond. However, the C-C bonds are having same strength of electron localization as in case of pristine T6 structure. For Li intercalated Si doped T6 system, small changes in isovalue around the bonding region between C atoms i.e., 0.78 and more importantly, no electron localization is found around any Li atoms. We also performed the electron localization function (ELF) study for B, N, Sn & Ge doped T6 and Li intercalated B, N, Sn & Ge doped T6 structure (shown in Fig. S9). In all these cases, similar results are observed. Only in case of Ge & Sn doped structures, destabilization around the doped element is easily visible. Over all, the shifting of Fermi level in DOS and less localization in the vicinity of Li atom are seen from ELF 2D plot confirm that the Li-C and Li-dopant bonds are ionic in nature.

### Computational Methods

All our calculations are performed within the frame work of DFT using the PWSCF code as distributed through the Quantum ESPRESSO package^30^. The exchange-correlation energy is calculated by using the generalised gradient approximation (GGA) as implemented by Perdew, Burke, and Ernzerhof (PBE)^31^. The Broyden-Fletcher-Goldfarb-Shanno (BFGS) method is used for geometry optimization. Atomic positions are fully relaxed in all calculations in which the Hellmann-Feynman forces converge within limit of 0.01 eV/Å. The Kohn-Sham orbitals are represented by the plane-wave basis set with the kinetic energy cut-off of 30 Ry (or 408 eV). For the integration over the Brillouin zone, the Monkhorst and Pack grid of (3 × 5 × 1) and (5 × 5 × 3) are used for the T6 (100) and T6 bulk structure, respectively and Methfessel-Paxton first-order spreading technique is used with a smearing width of 0.02 Ry (or 0.3 eV).

## Conclusions

Our studies of Li adsorption on T6 (100) surface showed that Li adsorption are highly preferable at C-B and C-H sites, because of strong interaction between s-orbital with σ and π orbitals. The adsorption of Li atoms on T6 (100) are stronger than pristine graphene. The potential energy plot, charge density and PDOS results bear the all above arguments. From the energy barrier calculations, it is found that Li diffusion across the plane of Si doped T6 (100) is easy with low energy barrier. The Si doped T6 structure is (Si_0.25_C_5.75_) proved to show better performance than pristine T6 during Li intercalation in terms of ΔE_f_, volume expansion and OCV. Therefore, we can tune inactive T6 structure into an excellent Li storage material by doping with neutral dopant (Si). The bonding nature of Li with Si and C are ionic in character. The Li atoms can be well dispersed in the Si doped T6 structure with the conformation of Li_1.7_Si_1_C_5_, specific capacity of about 632 mAh/g and volume expansion of only about 12%. Overall, with enhanced performance such as higher specific capacity, longer life time and safety, Si doped T6 structure (a mixed sp^2^ and sp^3^ hybridized material) can become a good an anode material for LIB’s.

## Additional Information

**How to cite this article**: Rajkamal, A. *et al*. Si doped T6 carbon structure as an anode material for Li-ion batteries: An *ab initio* study. *Sci. Rep*. **6**, 37822; doi: 10.1038/srep37822 (2016).

**Publisher's note:** Springer Nature remains neutral with regard to jurisdictional claims in published maps and institutional affiliations.

## Supplementary Material

Supplementary Information

## Figures and Tables

**Table 1:** Adsorption energies E_ad_ of a Li atom adsorbed on various sites of T6 (100) surface.

Sites on T6 (100)	ΔE_ad_ (eV)	d (Å)	ΔE (eV)
Atop	−1.02	2.16	1.7
C-C Bridge	−1.04	2.15	1.7
Hex-Hollow	−1.08	1.94	1.7
Centre-Bridge	−1.51	1.56	2.0
Centre-Hollow	−1.54	1.36	2.6

ΔE represents shift of ‘s’ orbital peak towards higher energy level of Li atom after adsorption on the surface. Here ‘d’ denotes the optimized distance between Li atom and T6 (100) surface.

**Table 2:** Common carbon based anode materials used for LIB’s and their theoretical specific capacities.

Anode materials	Conformation	Specific capacity (mAh/g)
Lithium^1^,^8^	_—_	3860
SWCNT^24^,^27^,^28^	LiC_2_	1116
Graphenylene^19^	Li_3_C_6_/Li_2.5_C_6_	1116/930
Graphite^9^	LiC_6_	372
Graphyne^18^	Li_1.5_C_6_	558
Si doped T6^T.W^	Li_1.7_Si_1_C_5_	632

This work is denoted with T. W.
