# Supplementary material for: Si doped T6 carbon structure as an anode material for Li-ion batteries: An ab initio study
Source: Sci Rep. 2016 Nov 28;6:37822. doi: 10.1038/srep37822 (PMC5124951; doi:10.1038/srep37822)
Supplement: Supplementary Information [file srep37822-s1.pdf]

Supplementary Information

Si doped T6 carbon structure as an anode material for Li-ion batteries: An *ab initio* study

A. Rajkamal,[1,#] E. Mathan Kumar,[2,#] V. Kathirvel[1], Noejung Park**,[3] and Ranjit Thapa*[1,2]

[1] Department of Physics and Nanotechnology, SRM University, Kattankulathur 603203, Tamil Nadu, India

[2]SRM Research Institute, SRM University, Kattankulathur 603203, Tamil Nadu, India

[3]Department of Physics, Center for Multidimensional Carbon Materials, Institute for Basic Science (IBS), Ulsan 689-798, Republic of Korea

* Corresponding Author E-mail: ranjit.t@res.srmuniv.ac.in, ranjit.phy@gmail.com; Tel. No.: +91-44-27417918, Fax: +91-44-27456702

** Corresponding Author Email: noejung@unist.ac.kr

[#]Same Contributed Authors

Contain figures represent the top and side view of T6 (001) surface, adsorption sites of Li atom on T6 (100) surface, diffusion of Li atom across (100) plane of both pristine and Si doped T6 structure. Structures demonstrating the sites chosen during Li intercalation in the Si doped T6 structure. Average OCV value of Si doped T6 structures is demonstrated by considering bulk material (without any Li intercalation) as reference. Atomic structures of Li intercalated (a) B, (b) N, (c) Sn, (d) Ge doped T6 are shown. Estimated values of Formation energy, OCV, and Volume expansion for B, N, Sn and Ge doped T6 are shown. Density of states (DOS) and ELF for Li intercalated and merely B, N, Sn, Ge doped T6 are demonstrated. Theoretical specific capacity calculation for graphite, SWCNT and Si doped T6 structures are given in details.

Content:

Fig. S1: Top and side view of T6 (001) surface.

Fig. S2: Adsorption sites of Li atom on T6 (100) surface.

Fig. S3: Diffusion of Li atom across (100) plane of both pristine and Si doped T6 structure.

Fig. S4: Structures demonstrating the sites chosen during Li intercalation in the Si doped T6 structure.

Fig. S5: Average OCV value of Si doped T6 structures are demonstrated by considering bulk material (without any Li intercalation) as reference.

Fig. S6: Atomic structure of Li intercalated (a) B, (b) N, (c) Sn, (d) Ge doped T6 structure.

Fig. S7: Estimated value of Formation Energy ΔE_f, OCV, and Volume expansion for B, N, Sn, Ge and Si doped T6 structure.

Fig. S8: Density of states (DOS) for Li intercalated and merely B, N, Sn, Ge doped T6 structure.

Fig. S9: Electron localisation function (ELF) plot for both Li intercalated and de-intercalated B, N, Sn, Ge doped T6 structure.

Theoretical Specific capacity calculations.

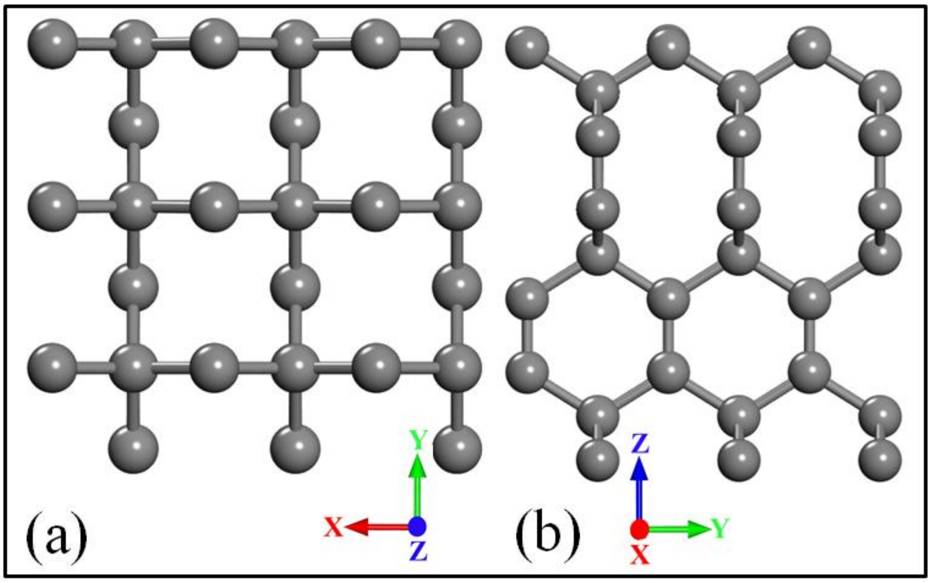

Figure S1: (a) Top and (b) side view of the (3×3×1) T6 (001) plane. The grey balls represent the C atoms.

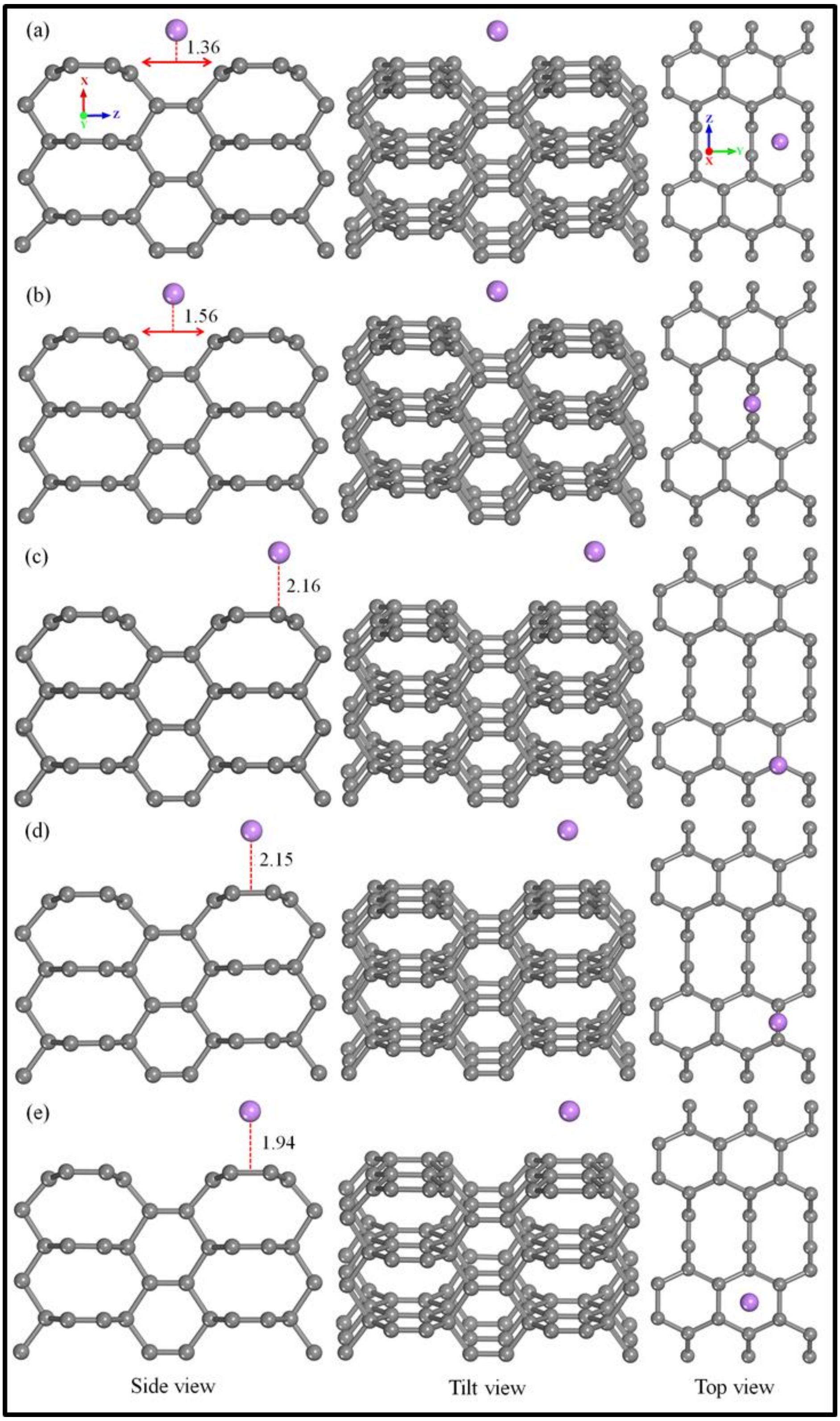

Figure S2: (a-e) Side, Tilt and Top view of Li atom adsorbed on five different sites, namely C-H, C-B, A, B and H of T6 (100) surface respectively. The grey and violet balls denote the C and Li atom respectively.

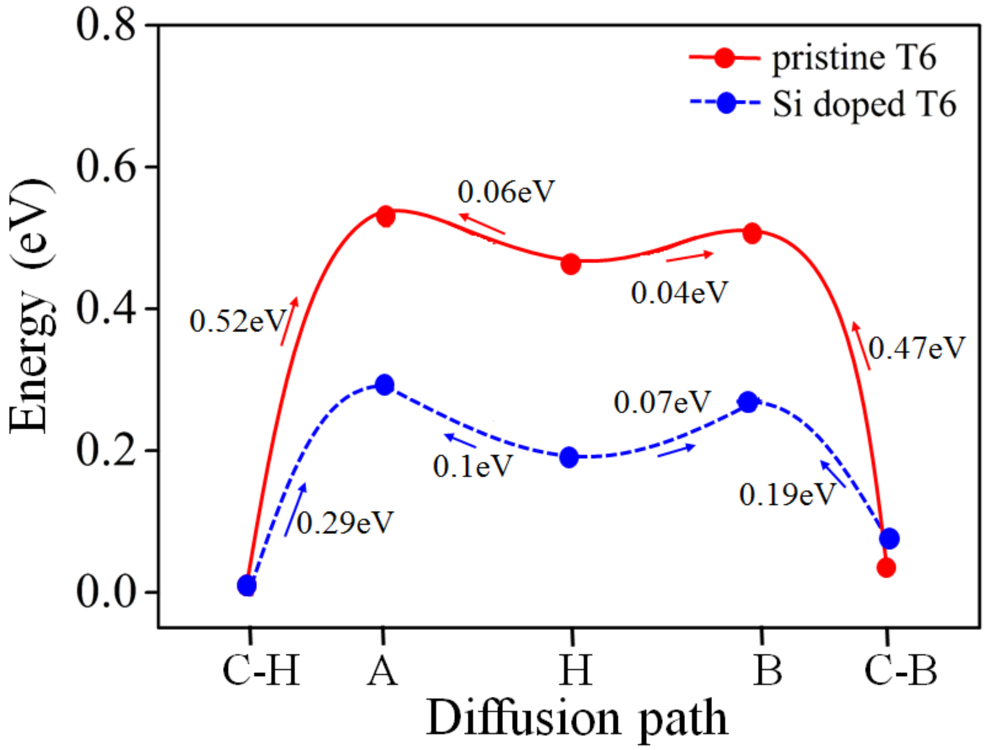

Figure S3: The energy barrier against Li diffusion along various paths on the pristine T6 (solid red line) and Si doped T6 (100) surface (dashed blue line). C-H, A, H, B and C-B represents Centre-Hollow, Atop, Hex-Hollow, C-C Bridge and Centre-Bridge respectively. Arrows (blue and red) indicates the direction of migration.

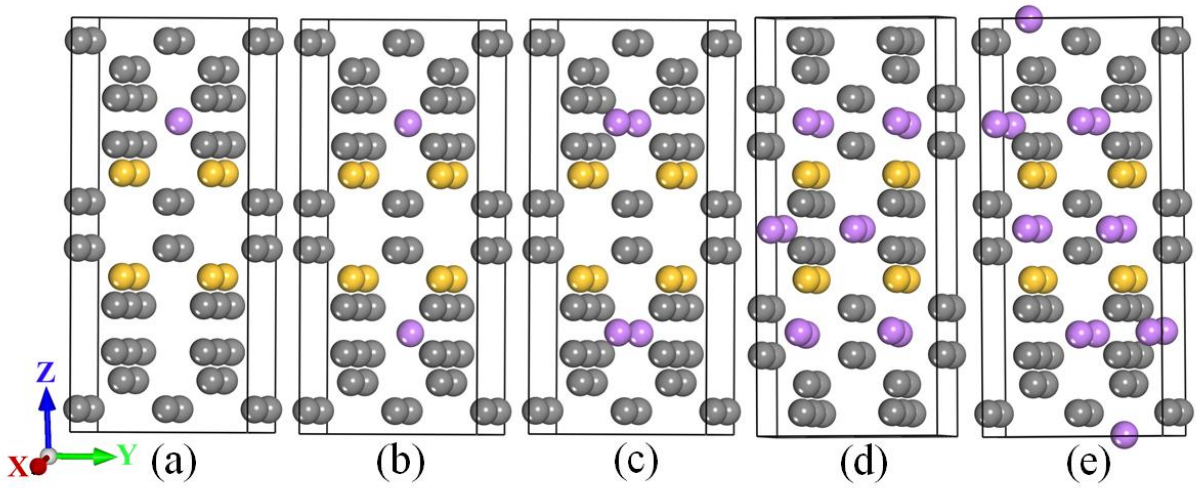

Figure S4: Li-intercalated configuration of Si doped T6 structures with various concentrations. (a) One Li (b) two Li (c) four Li (d) twelve Li and (e) fourteen Li intercalated systems. Grey, yellow and violet spheres are representing C, Si and Li atoms respectively.

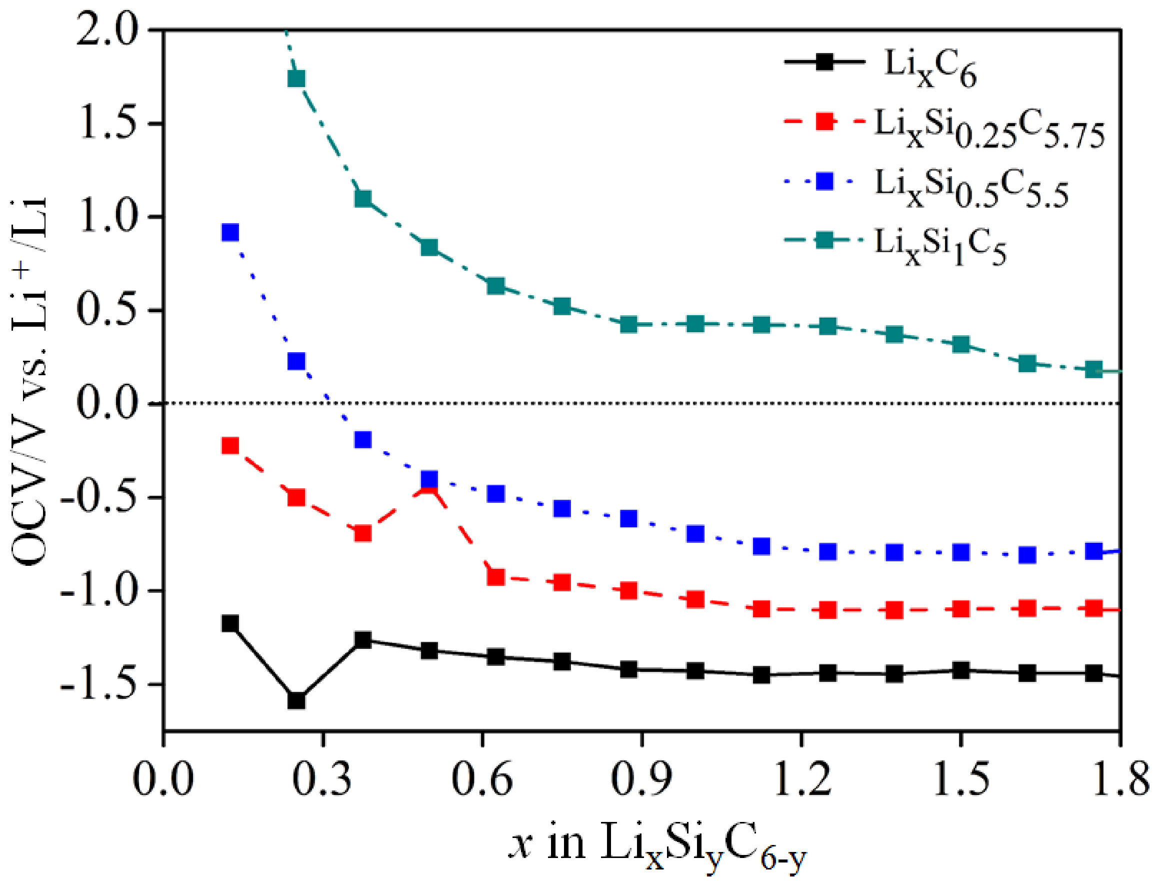

Figure S5: Demonstrate the calculated OCV values for the Si doped T6 ($Li_xSi_yC_{6-y}$) as a function of the Li (x) and Si (y) concentration. The lines serve as guides to the eye. The horizontal dashed line is minimum frontier line for OCV.

Here (**only for this Figure**), we examine the average open circuit voltage (OCV) defined as

$$\mathrm{OCV}\ (x) = [E_C + nE_{Li}^{bulk} - E_{C-Li}^{x}]\ /ne\ ,$$

where E_{Li}^{bulk} is the total energy of single Li atom of bulk bcc lithium, E_C is the total energy of the anode material (without Li intercalated) and E_{C-Li}^{x} is the total energy of Li intercalated system for a given x = 6(n/n_c), n is the number of Li atoms intercalated in the compound, e is the charge of electron, n_c is the total number of carbon atom per supercell. In simple word the average OCV value of Si doped T6 structures is calculated by considering bulk material (without any Li intercalation) as reference.

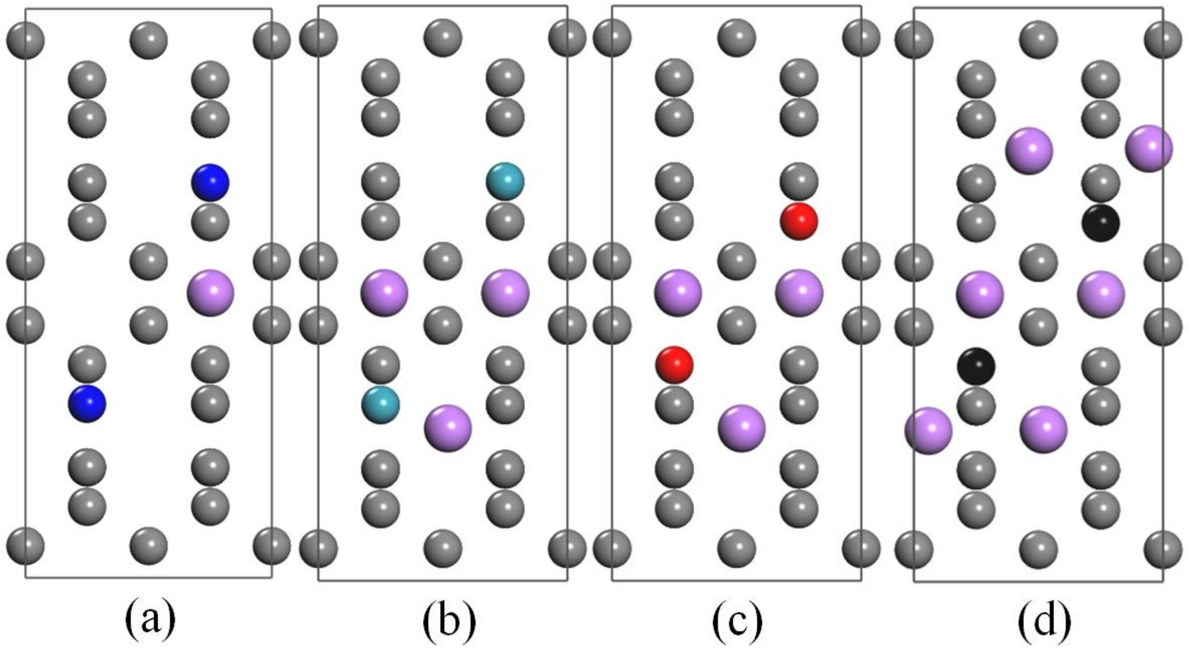

Figure S6: Atomic structure of Li intercalated (a) B, (b) N, (c) Sn, (d) Ge doped T6 structure. Blue, cyan, red, black, grey and violet spheres are interposing B, N, Sn, Ge, C, and Li atoms respectively.

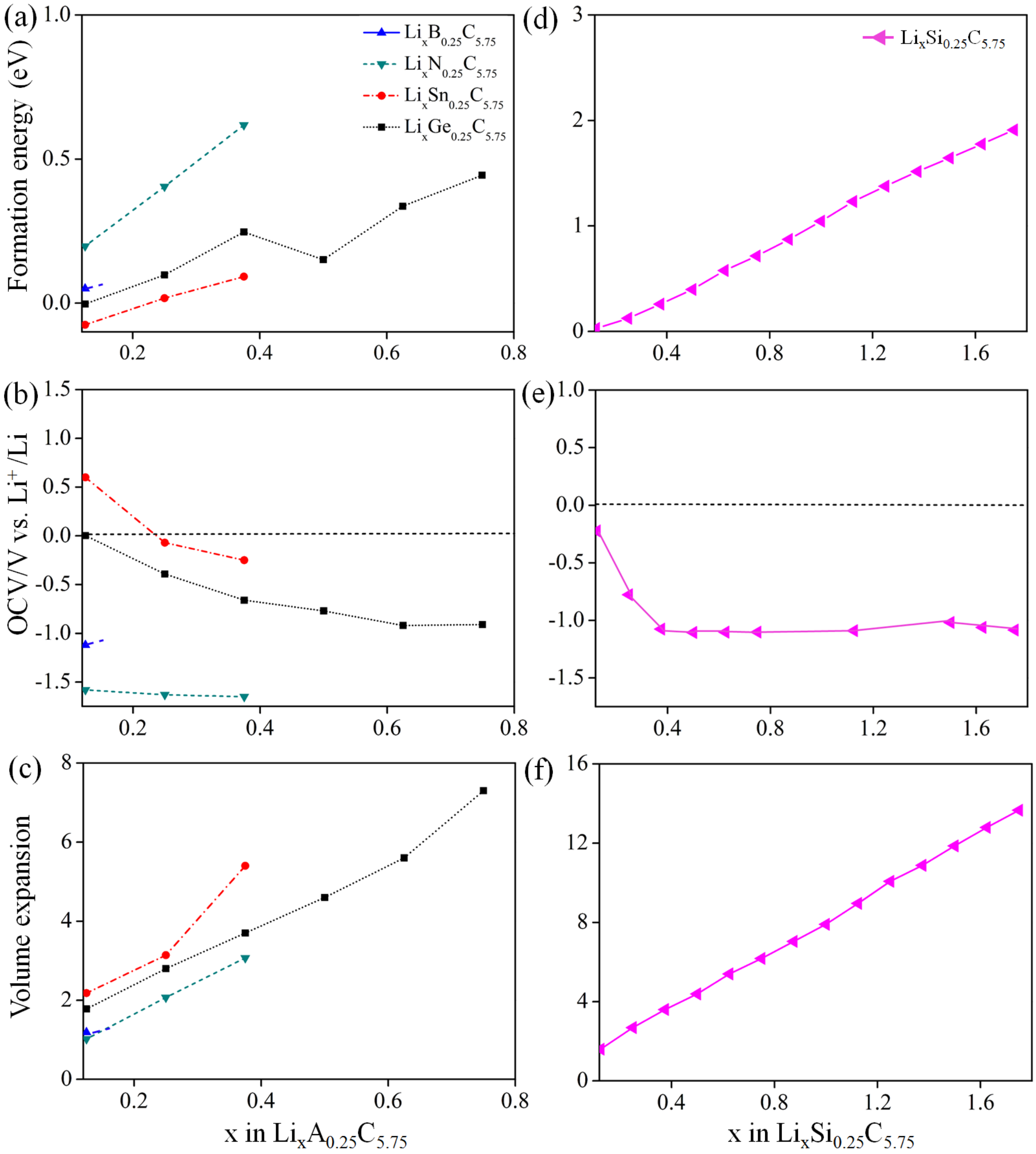

Figure S7: (a, d) Formation Energy ΔE_f as defined in the main text, (b, e) OCV, (c, f) Volume expansion as a function of the Li (x) concentration for B, N, Sn, Ge and Si doped T6 structure as a function of concentration of Li intercalation. Here A denotes the element B, N, Sn and Ge. The lines serve as guides to the eye. In (b) and (e) the horizontal dashed line is minimum frontier line for OCV.

For OCV calculation (in Figure S7), we followed the formula same as in the main text.

$$\mathrm{OCV} = \left[E\left(\mathrm{Li}_{x_2}C_6\right) - E\left(\mathrm{Li}_{x_1}C_6\right) - (x_2 - x_1)E(\mathrm{Li})\right] / (x_2 - x_1)\,F\,,$$

where $E\left(\mathrm{Li}_{x_2}C_6\right)$ and $E\left(\mathrm{Li}_{x_1}C_6\right)$ are the total energy of the compound with the Li concentrations of x_2 and x_1, respectively and F is the Faraday's constant. We consider OCV at the midpoint x i.e. $(x_1 + x_2)$ / 2 in which OCV is calculated at interval between x_1 and x_2.

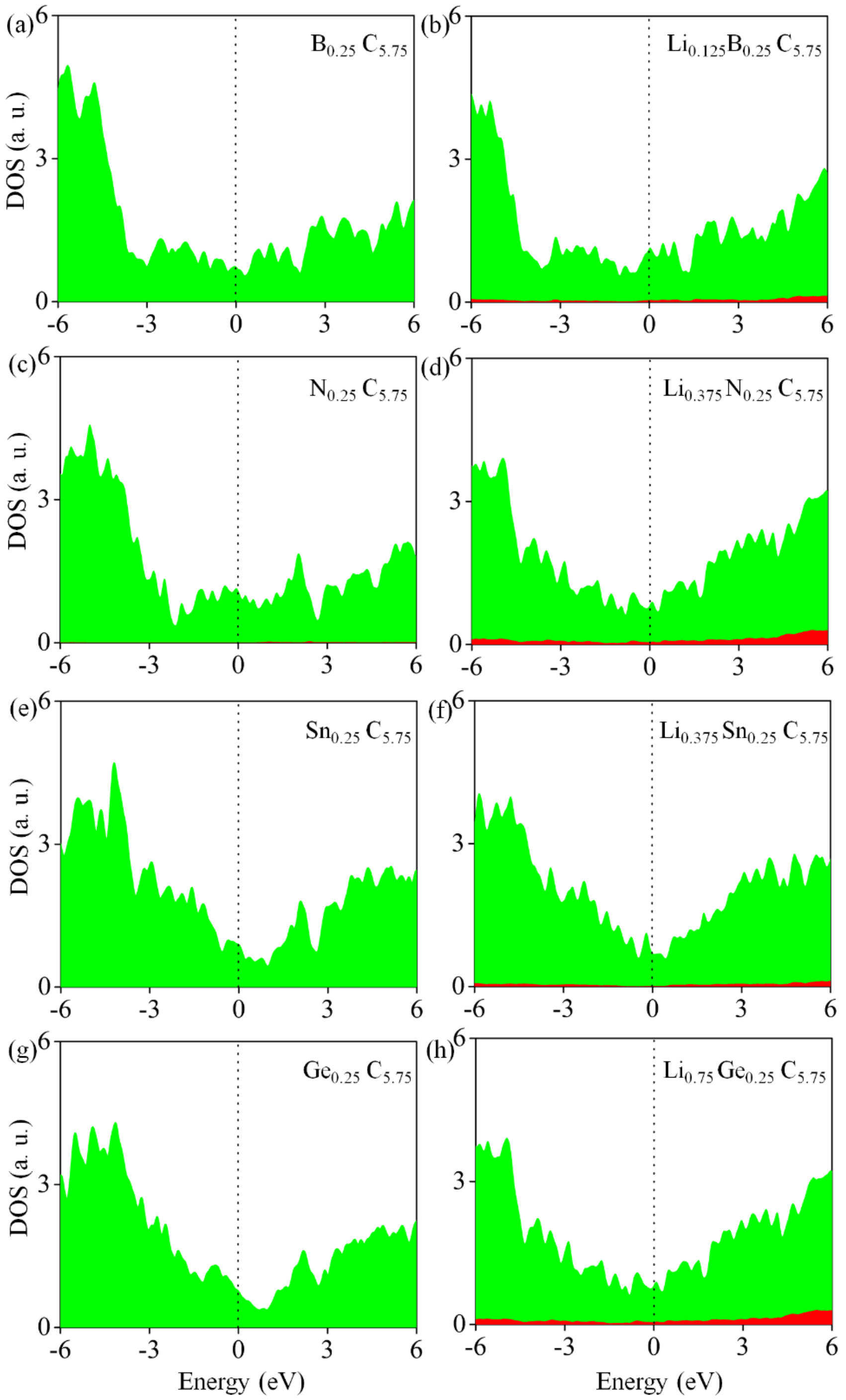

Figure S8: Density of states (DOS) for (a) B, (c) N, (e) Sn, (g) Ge doped T6 structure. (b), (d), (f), and (h) depicts the density of states for Li intercalated (B, N, Sn and Ge) doped T6 systems. (a-h) Filled green area indicates DOS for 'p' states of C atoms, and in (b, d, f and h) filled red area represents the DOS for 's' states of Li atoms. The dashed lines indicate the position of Fermi level.

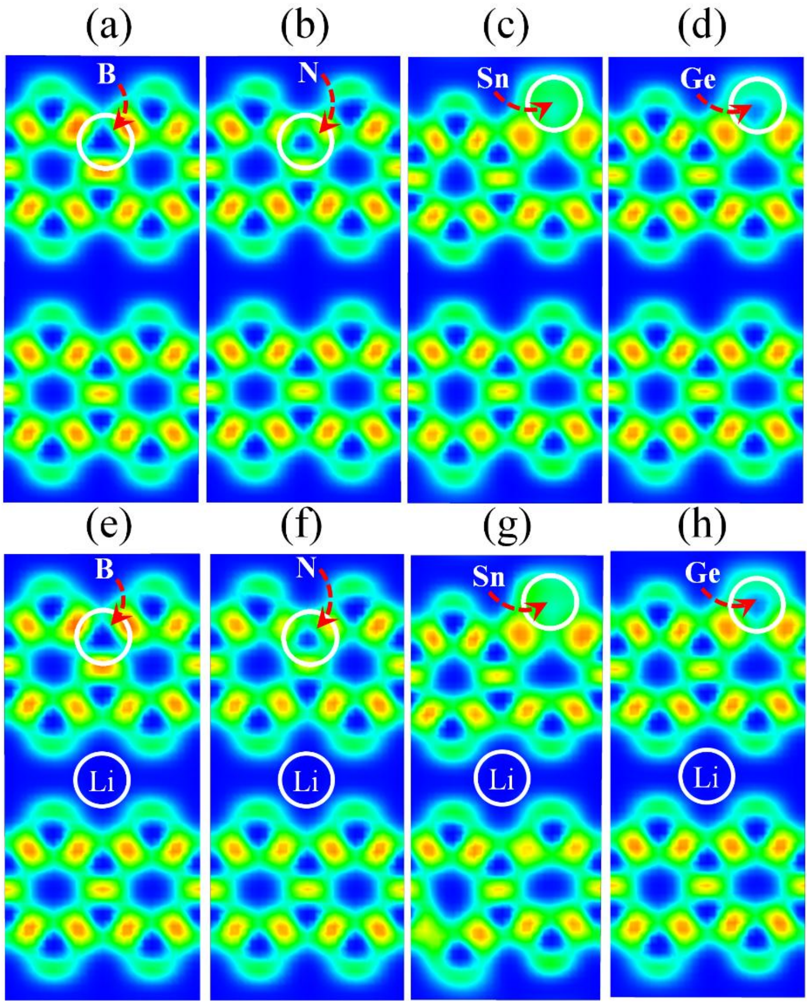

Figure S9: ELF 2D plot for (a) B, (b) N, (c) Sn and (d) Ge doped T6 structure and Li intercalated (e) B, (f) N, (g) Sn and (h) Ge doped T6 structure (the atomic structures are shown in Fig. S6). The positions of atoms are circled with white colour and it is indicated by red dashed arrow.

The density of states of B, N, Sn and Ge doped T6 structure is shown in Figure S8. All these doping consistently produces the metallic electronic structures. In this case also the Li-C and Li-dopant bond is ionic in nature. To verify the nature of the bond we investigated the electron localization function (ELF) for B, N, Sn & Ge doped T6 and also for Li-intercalated B, N, Sn & Ge doped T6 structure. The covalent interaction between C-C and dopant atoms

with C are more clearly illustrated from the ELF plot shown in the Fig. S9 (a-h). In all cases, sp^2 and sp^3 bonded C atoms (C-C) are surrounded by high localization with the value of around 0.78 to 0.85. This clearly indicates that C atoms are bonded covalently (denoted by red-yellow contours) and the pore regions have less localization (indicated by blue contours). On the other hand, in case of Ge & Sn doped structures, destabilization around the doped element is easily visible. This indicates, the stability is largely reduced between the Sn-C and Ge-C bonds. However, the C-C bonds are having almost same strength of electron localization as in case of pristine T6 structure. For Li intercalated B, N, Sn & Ge doped T6 system, small changes in isovalue around the bonding region between C atoms i.e., 0.76 to 0.82. It is noted that no electron localization is found around any Li atoms. This data confirms again, Li-C and Li-dopant bonds are also ionic in nature.

Theoretical Specific capacity (Sp .cap) calculations:

Specific Theoretical capacity (mAh/g) = $[(F \times n_{Li}) / (M \times 3600)] \times 1000$.

Where,

F = Faraday's constant (96,500 coulombs per gm equivalent).

n_{Li} = Number of Li per formula (Li_xC_6) unit of the electrode material.

$n_{Li} = x = 6 (n/n_c)$

n = no of lithium atom adsorbed in the anode material.

n_c = no of carbon atom in the anode material.

M = Molecular mass of the electrode material.

Other way,

Specific Theoretical capacity (mAh/g) = $(n_{Li}$ x 26.801 x 1000)/M.

26.801 = Faradays constant / (Charging battery /1 hour)

= 96,500 / (3600).

If this has to be expressed in terms of current, divide that by 3600

F = 26.801Ah/Mole

M = molecular weight

For mA we multiply with 1000.

1. Graphite anode material for LiC_6:

$n_{Li} = x = 6\ (n/n_c)$

$= 6\ (4/24)$

$= 1$

Sp. cap = (1 x 26.801 x 1000)/ (6x12.011) = 372 mAh/g.

Six C atoms molecular weight = 6 x 12.011.

2. SWCNT anode material for LiC_2:

Sp. cap = (1 x 26.801 x 1000)/ (2x12.011) = 1116 mAh/g.

3. Si doped T6 anode material can have conformation of $Li_{1.7}Si_1C_5$:

Sp. cap = (1.7 x 26.801 x 1000)/ (6x12.011) = 632 mAh/g.
